# A Case of Idiopathic Orbital Inflammation in a Seven-Year-Old Child

**DOI:** 10.7759/cureus.13924

**Published:** 2021-03-16

**Authors:** Basma Alqaseer, Isa Almutawa

**Affiliations:** 1 Ophthalmology, Bahrain Defence Force Royal Medical Services (BDFRMS), Riffa, BHR

**Keywords:** inflammation, pediatrics, ioi, pseudotumor

## Abstract

We present a case of idiopathic orbital inflammation (IOI) in a pediatric patient that was initially diagnosed as orbital cellulitis. A seven-year-old female presented with painful right upper eyelid swelling and ptosis and an upper respiratory tract infection. Ruling out secondary causes that are sight and life-threatening conditions is prudent. Therefore, a multidisciplinary approach is crucial in the diagnosis and management of IOI. Consulting with different specialties aids in diagnosing and managing the condition, and thus optimizing patient care.

## Introduction

Idiopathic orbital inflammation (IOI), also known as orbital pseudotumor, is a benign, non-infectious inflammatory lesion of unknown etiology [1,2]. IOI is considered to be the third most common cause of unilateral proptosis in adults between the third and sixth decade, following thyroid eye disease and lymphoproliferative disease [1,3]. Patients typically present with symptoms of orbital pain with signs of swelling, proptosis, ophthalmoplegia, and variable change in vision [1,4].

IOI is rarely seen in children [1,3-5]. Only approximately 70 cases have been reported [1]. Therefore, only little is known about the condition in the pediatric age group [1,3]. Children most commonly present with a palpable mass, pain, proptosis, eyelid edema, restricted extraocular movements, and increased intraocular pressure (IOP) [1]. Affected children are at risk of recurrence and may have bilateral involvement and constitutional signs, such as headache, fever, lethargy, vomiting, and abdominal pain [1,5-8].

IOI is a diagnosis of exclusion [8]. It is crucial to rule out secondary causes of orbital inflammation and proptosis in children, such as orbital cellulitis, rhabdomyosarcoma, leaking dermoid cyst, thyroid-associated orbitopathy (TAO), leukemia, lymphoma, and histiocytosis X [1,2,4,6,8,9].

In addition to thorough history and examination, radiological investigations aid in establishing a diagnosis [1,2,4]. Imaging shows thickening of extraocular muscles and their tendons (in myositis), involvement of orbital fat and lacrimal gland (in dacroadenitis), and involvement of the posterior sclera (in posterior scleritis) described as “ring sign,” and with clear sinuses [6]. Laboratory tests are often normal, except for elevated erythrocyte sedimentation rate (ESR) and eosinophilia [5,9]. Children usually respond well to corticosteroid therapy [4]. Biopsy is only reserved for refractory or atypical conditions [2,4,8].

We present a case of an IOI in a pediatric patient that was initially diagnosed as orbital cellulitis.

## Case presentation

**Figure 1:**
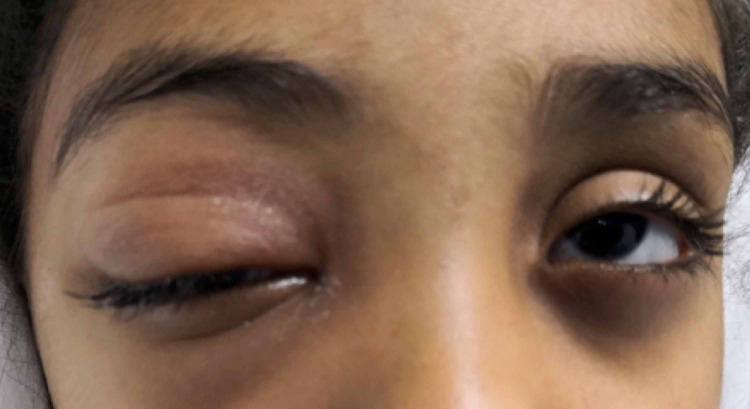
Clinical photograph of the child on presentation showing right eye ptosis.

A seven-year-old female presented with painful right upper eyelid swelling and ptosis, associated with lethargy, nausea, and vomiting (Figure 1). The child had a history of upper respiratory tract infection without fever. No history of trauma preceded the presentation. She was diagnosed with preseptal cellulitis by a general practitioner at a different hospital and commenced on oral paracetamol and antibiotics (amoxicillin/clavulanic acid).

The patient was afebrile and vital parameters were stable. Ophthalmic examination showed right upper eyelid edema, tenderness, and ptosis. Limited elevation and abduction of the right eye were noted. Visual acuity was 20/25 bilaterally; anterior segment and dilated fundus examinations were both within normal limits. IOP could not be assessed.

**Figure 2:**
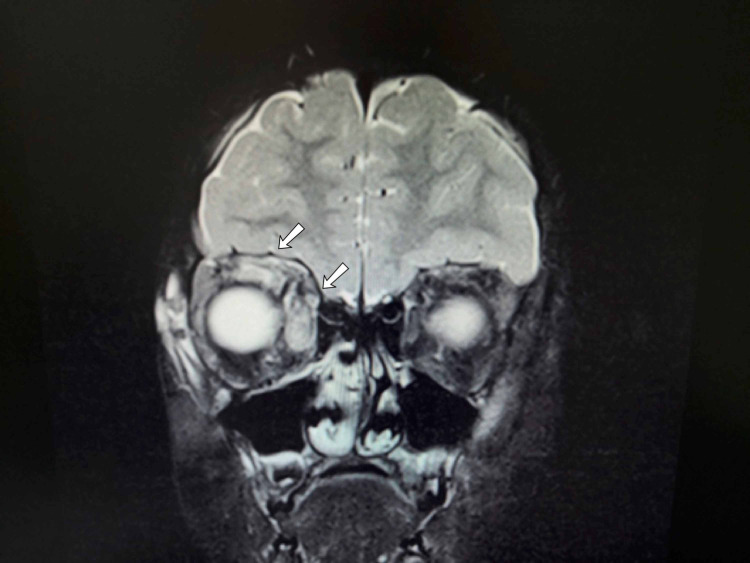
Coronal MRI orbits with gadolinium showing enhancing, thickened right superior and medial rectus muscles with tendon involvement. MRI, magnetic resonance imaging

The patient was admitted to rule out orbital cellulitis and commenced on intravenous (IV) fluid and IV antibiotics: clindamycin and ceftriaxone. After 48 hours, the patient’s condition neither improved nor deteriorated. Computed tomography (CT) imaging was done and referral to otolaryngology team was made to rule out orbital cellulitis secondary to acute sinusitis. Imaging showed right preseptal soft tissue swelling and exophthalmos, thereby excluding a diagnosis of orbital cellulitis and acute sinusitis as a secondary cause. Laboratory investigations were normal, except high ESR. Orbital magnetic resonance imaging (MRI) showed thickening of the right superior and medial rectus muscles with tendon involvement and diffusely enlarged lacrimal gland (Figure 2).

**Figure 3:**
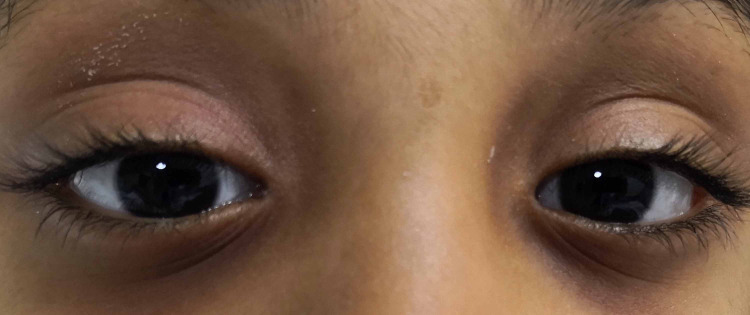
Clinical photograph of the child at the time of discharge showing resolution of right eye ptosis.

The patient was started on IV corticosteroid at 1.5 mg/kg/day with gastric prophylaxis after consulting with pediatrics. A drastic improvement was noted after 24 hours. Antibiotics were discontinued and the child was discharged home after receiving three days of IV corticosteroids. Treatment was continued as an outpatient on oral corticosteroids at 1.5 mg/kg/day and gradually tapered over six weeks. Complete resolution of symptoms was noted at the last follow-up six weeks from discharge (Figure 3).

## Discussion

IOI is less common in the pediatric age group than in adults. Compared to adults, pediatric IOI is more commonly associated with bilateral involvement and constitutional signs [1,8]. Only one-third of cases in the pediatric age group have bilateral presentation [7-9]. Constitutional signs occur in about 50% of cases [1,8]. In our case, the child presented with constitutional signs and unilateral involvement.

The most common clinical features of IOI are periorbital edema, followed by pain and ptosis [10]. The cause of IOI remains unknown. Several theories suggest an autoimmune disorder, upper respiratory infections, and minor trauma as triggers [1,5,8]. In our case, the child had a history of upper respiratory tract infection. However, most studies described this condition as idiopathic [1,5,8]. IOI is considered a diagnosis of exclusion; therefore, a thorough evaluation of the differential diagnosis is paramount [1,8,9]. In the pediatric population, it is crucial to rule out orbital cellulitis, rhabdomyosarcoma, leaking dermoid cyst, TAO, leukemia, lymphoma, and histiocytosis X [1,2,4,6,8,9].

IOI is often misdiagnosed as orbital cellulitis [5]. A study that involved 30 cases of IOI in a pediatric age group showed that 50% of the cases were misdiagnosed by their initial healthcare provider, ranging from pediatricians, emergency physicians, and general ophthalmologists [1]. Patients with orbital cellulitis can present with similar symptoms to IOI, such as pain, proptosis, ophthalmoplegia, and periorbital edema [1,11]. Prompt intervention of orbital cellulitis is crucial as it is a potentially sight and life-threatening condition [11]. Fever may be absent in some cases of orbital cellulitis [12]. Systemic antibiotics should be administered first for 48 hours in cases where it is difficult to differentiate. If there are no signs of improvement following the administration of systemic antibiotics within 48 hours, it would rule out orbital cellulitis [3]. This was seen in our case. The child was admitted with suspected orbital cellulitis secondary to suspected acute sinusitis. She was started on systemic antibiotics, but did not show any improvement.

TAO and IOI can both result in orbital myositis. On imaging, the extraocular muscle inflammation in TAO is associated with sparing of tendons, while in IOI the tendons are involved [8]. Although IOI can affect any orbital soft tissue, the most common structures affected are the rectus muscles and lacrimal gland [1]. Our patient’s MRI showed thickening of the right superior and medial rectus muscles with tendon involvement, along with lacrimal gland enlargement, and thus not favoring a diagnosis of TAO.

Laboratory tests in IOI reveal both eosinophilia and high ESR [5,9]. In our case, ESR was high, but without eosinophilia. A diagnosis of IOI was made in our case based on the clinical examination, imaging, and improvement following IV corticosteroid without biopsy. Biopsy and histological confirmation are usually reserved for cases that are refractory, show progression, and are recurrent or atypical despite corticosteroid treatment [2,4,8].

IOI is a benign condition; however, treatment is crucial as it is sight-threatening. Systemic corticosteroid therapy is the first-line treatment, with oral corticosteroid being the mainstay treatment [1,2]. However, in our case, as the patient was not complying with oral corticosteroid therapy, she was commenced on IV corticosteroid. A quick response to corticosteroids is considered diagnostic [3,5]. Improvement in our case was noted within the first 24 hours of treatment. Pediatric dosage is usually started and maintained at 1-1.5 mg/kg/day [5,6,8]. Maintenance dose is started at oral prednisone at 1-1.5 mg/kg/day and gradually tapered over six to eight weeks [2].

In children, resolution of symptoms usually occurs within 24 to 48 hours [3,5]. If corticosteroid is medically contraindicated, there is poor response to corticosteroid, or if it recurred despite corticosteroid therapy, then local radiotherapy at low dose is considered [3,5,6]. Radiotherapy should only be considered if other causes have been excluded [6].

A multidisciplinary approach is crucial in the management of IOI. Consulting with different specialties, such as otolaryngologist, pediatricians, and radiologist, aids in diagnosing and managing the condition, and thus optimizing patient care [1,8].

## Conclusions

IOI is a rare, benign, non-infectious inflammatory condition. As it is a diagnosis of exclusion, it is imperative to rule out other pathological and sight-threatening conditions. Therefore, careful history taking, thorough examination, relevant investigations, and multidisciplinary care are crucial steps in the management of IOI.
